# Diaqua­bis(tetra­zolo[1,5-*a*]pyridine-8-carboxyl­ato-κ^2^ *N* ^1^,*O*)cobalt(II) dihydrate

**DOI:** 10.1107/S1600536809019187

**Published:** 2009-05-23

**Authors:** Min Xue, Fu-Chen Liu

**Affiliations:** a School of Chemistry and Chemical Engineering, Tianjin University of Technology, Tianjin 300191, People’s Republic of China

## Abstract

In the title compound, [Co(C_6_H_3_N_4_O_2_)_2_(H_2_O)_2_]·2H_2_O, the Co^II^ atom is located on an inversion center in a slightly distorted octa­hedral environment formed by the O atoms of two water mol­ecules, and the N and O atoms of the chelating tetra­zolo[1,5-*a*]pyridine-8-carboxyl­ate anions. Hydrogen bonds of the O—H⋯O and O—H⋯N types result in a three-dimensional supra­molecular network.

## Related literature

For background to coordination compounds and their synthesis by *in situ* reaction, see: Chen & Tong (2007 ▶); Liu *et al.* (2005 ▶); Li *et al.* (2007 ▶).
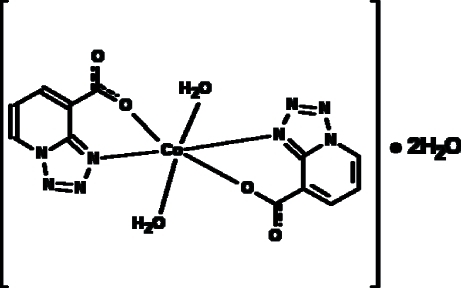


## Experimental

### 

#### Crystal data

[Co(C_6_H_3_N_4_O_2_)_2_(H_2_O)_2_]·2H_2_O
*M* *_r_* = 457.24
Orthorhombic, 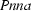

*a* = 19.041 (4) Å
*b* = 11.694 (2) Å
*c* = 7.5371 (15) Å
*V* = 1678.3 (6) Å^3^
*Z* = 4
Mo *K*α radiation
μ = 1.09 mm^−1^
*T* = 293 K
0.5 × 0.5 × 0.4 mm

#### Data collection

Rigaku SCXmini diffractometer
Absorption correction: multi-scan (*ABSCOR*; Higashi, 1995 ▶) *T*_min_ = 0.530, *T*_max_ = 0.667
13120 measured reflections
1482 independent reflections
1203 reflections with *I* > 2σ(*I*)
*R*_int_ = 0.081

#### Refinement

*R*[*F* ^2^ > 2σ(*F* ^2^)] = 0.055
*wR*(*F* ^2^) = 0.090
*S* = 1.21
1482 reflections
148 parameters
H atoms treated by a mixture of independent and constrained refinement
Δρ_max_ = 0.34 e Å^−3^
Δρ_min_ = −0.54 e Å^−3^

### 

Data collection: *SCXmini Benchtop Crystallography System Software* (Rigaku, 2006 ▶); cell refinement: *PROCESS-AUTO* (Rigaku, 1998 ▶); data reduction: *PROCESS-AUTO*; program(s) used to solve structure: *SHELXS97* (Sheldrick, 2008 ▶); program(s) used to refine structure: *SHELXL97* (Sheldrick, 2008 ▶); molecular graphics: *ORTEPIII* (Burnett & Johnson, 1996 ▶) and *PLATON* (Spek, 2009 ▶); software used to prepare material for publication: *SHELXTL* (Sheldrick, 2008 ▶).

## Supplementary Material

Crystal structure: contains datablocks global, I. DOI: 10.1107/S1600536809019187/ng2582sup1.cif

Structure factors: contains datablocks I. DOI: 10.1107/S1600536809019187/ng2582Isup2.hkl

Additional supplementary materials:  crystallographic information; 3D view; checkCIF report

## Figures and Tables

**Table 1:** Hydrogen-bond geometry (Å, °)

*D*—H⋯*A*	*D*—H	H⋯*A*	*D*⋯*A*	*D*—H⋯*A*
O1*W*—H1*WB*⋯O2^i^	0.825 (18)	1.950 (19)	2.763 (4)	168 (4)
O1*W*—H1*WA*⋯O2*W*^ii^	0.842 (19)	1.943 (19)	2.776 (5)	170 (5)
O2*W*—H2*WB*⋯O1	0.835 (19)	2.04 (3)	2.845 (4)	163 (4)
O2*W*—H2*WA*⋯N2^iii^	0.842 (19)	2.15 (2)	2.981 (5)	171 (4)

Symmetry codes: (i) 
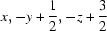
; (ii) 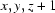; (iii) 
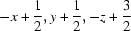
.

**Fig. 1.**
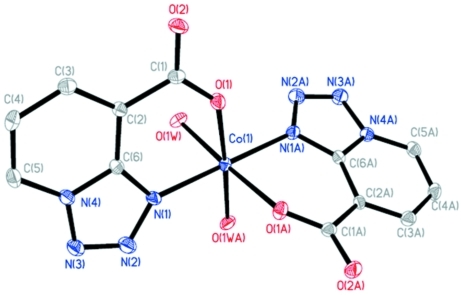
A view of the title compound showing the coordination of Co atom with the atom-labelling scheme. Ellipsoids are drawn at the 30% probability level. H atom have been omitted for clarity. [ Symmetry codes: (a)-x+1/2,-y,z].

**Fig. 2.**
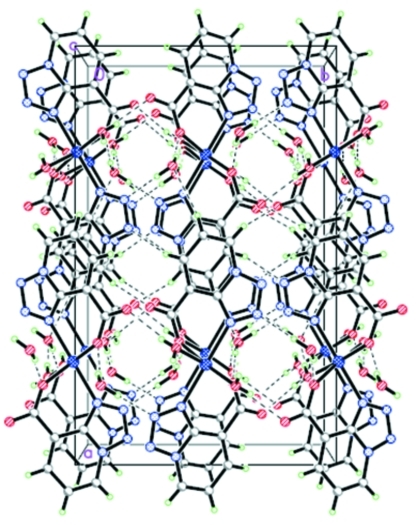
The 3D supramolecular net formed by the hydrogen bonds.

## supplementary crystallographic information

### Comment

Coordination complexes have attracted great attention in recent years. (Liu,*et al.*, 2005). The in-situ reaction which can create new ligand and structure draw much more attention in synthesizing coordination complexes (Li,*et al.*, 2007). Some interesting complexes were ganied by the in-situ reaction. (Chen,*et al.*, 2007).

In the title compound, the cobalt atom the cobalt atom located in the inverse center is six coordinated by two waters and two tetrazolo(1,5-*a*)pyridine-8-carboxylato, (Fig. 1). Each tetrazolo(1,5-*a*)pyridine-8-carboxylato chelates to one cobalt atom. One type of water coordinates to the cobalt and the other acts as lattice water. A three dimensional supramolecular net formed by the hydrogen bonds of waters and tetrazolo(1,5-*a*)pyridine-8-carboxylato ligands intermolecular (Fig. 2).

### Experimental

A mixture of cobalt(II)nitrate and sodium azide (1 mmol), 2-chloronicotinic acid(0.5 mmol), in 10 ml of water was sealed in a Teflon-lined stainless-steel Parr bomb that was heated at 363 K for 48 h. Red crystals of the title complex were collected after the bomb was allowed to cool to room temperature.Yield 20% based on cobalt(II). Caution: Azides may be explosive. Although we have met no problems in this work, only a small amount of them should be prepared and handled with great caution.

### Refinement

Hydrogen atoms were included in calculated positions and treated as riding on their parent C atoms with C—H = 0.93Å and *U*_iso_(H) = 1.2*U*_eq_(C).Hydrogen atom of water were added by difference Fourier maps and refined with restrainated distance of O—H = 0.85Å with a error of 0.02Å, and the restrainated distance  of H—H = 1.35Å with a error of 0.01Å of the same water.

### Crystal data

**Table:**

[Co(C_6_H_3_N_4_O_2_)_2_(H_2_O)_2_]·2H_2_O	*D*_x_ = 1.810 Mg m^−^^3^
*M**_r_* = 457.24	Mo *K*α radiation, λ = 0.71073 Å
Orthorhombic, *P**n**n**a*	Cell parameters from 11987 reflections
*a* = 19.041 (4) Å	θ = 3.3–27.8°
*b* = 11.694 (2) Å	µ = 1.09 mm^−^^1^
*c* = 7.5371 (15) Å	*T* = 293 K
*V* = 1678.3 (6) Å^3^	Block, red
*Z* = 4	0.5 × 0.5 × 0.4 mm
*F*(000) = 932	

### Data collection

**Table:**

Rigaku SCXmini diffractometer	1482 independent reflections
Radiation source: fine-focus sealed tube	1203 reflections with *I* > 2σ(*I*)
graphite	*R*_int_ = 0.081
ω scans	θ_max_ = 25.0°, θ_min_ = 3.2°
Absorption correction: multi-scan (*ABSCOR*; Higashi, 1995)	*h* = −22→22
*T*_min_ = 0.530, *T*_max_ = 0.667	*k* = −13→13
13120 measured reflections	*l* = −8→8

### Refinement

**Table:**

Refinement on *F*^2^	Primary atom site location: structure-invariant direct methods
Least-squares matrix: full	Secondary atom site location: difference Fourier map
*R*[*F*^2^ > 2σ(*F*^2^)] = 0.055	Hydrogen site location: inferred from neighbouring sites
*wR*(*F*^2^) = 0.090	H atoms treated by a mixture of independent and constrained refinement
*S* = 1.21	*w* = 1/[σ^2^(*F*_o_^2^) + (0.0256*P*)^2^ + 2.1174*P*] where *P* = (*F*_o_^2^ + 2*F*_c_^2^)/3
1482 reflections	(Δ/σ)_max_ < 0.001
148 parameters	Δρ_max_ = 0.34 e Å^−^^3^
0 restraints	Δρ_min_ = −0.54 e Å^−^^3^

### Special details

**Table:**

Geometry. All esds (except the esd in the dihedral angle between two l.s. planes) are estimated using the full covariance matrix. The cell esds are taken into account individually in the estimation of esds in distances, angles and torsion angles; correlations between esds in cell parameters are only used when they are defined by crystal symmetry. An approximate (isotropic) treatment of cell esds is used for estimating esds involving l.s. planes.
Refinement. Refinement of *F*^2^ against ALL reflections. The weighted *R*-factor *wR* and goodness of fit *S* are based on *F*^2^, conventional *R*-factors *R* are based on *F*, with *F* set to zero for negative *F*^2^. The threshold expression of *F*^2^ > σ(*F*^2^) is used only for calculating *R*-factors(gt) *etc*. and is not relevant to the choice of reflections for refinement. *R*-factors based on *F*^2^ are statistically about twice as large as those based on *F*, and *R*- factors based on ALL data will be even larger.

### Fractional atomic coordinates and isotropic or equivalent isotropic displacement parameters (Å^2^)

**Table:**

	*x*	*y*	*z*	*U*_iso_*/*U*_eq_	
Co1	0.2500	0.0000	0.86713 (10)	0.0185 (3)	
O1	0.20211 (15)	0.1011 (2)	0.6721 (4)	0.0258 (7)	
O1W	0.20777 (17)	0.1069 (3)	1.0644 (4)	0.0282 (8)	
H1WB	0.187 (2)	0.167 (2)	1.037 (5)	0.028 (14)*	
H1WA	0.234 (2)	0.120 (4)	1.151 (5)	0.034 (16)*	
O2	0.12294 (15)	0.2126 (2)	0.5359 (4)	0.0310 (8)	
N1	0.15291 (19)	−0.0903 (3)	0.8639 (4)	0.0220 (8)	
N2	0.1335 (2)	−0.1860 (3)	0.9493 (5)	0.0307 (10)	
N3	0.0650 (2)	−0.2019 (3)	0.9438 (5)	0.0315 (10)	
N4	0.03825 (19)	−0.1111 (3)	0.8530 (5)	0.0236 (9)	
C1	0.1390 (2)	0.1307 (3)	0.6326 (5)	0.0220 (10)	
C2	0.0793 (2)	0.0595 (3)	0.7064 (6)	0.0199 (9)	
C3	0.0093 (2)	0.0832 (4)	0.6782 (6)	0.0251 (11)	
H3A	−0.0022	0.1500	0.6180	0.030*	
C4	−0.0468 (2)	0.0108 (3)	0.7364 (6)	0.0276 (11)	
H4A	−0.0933	0.0318	0.7165	0.033*	
C5	−0.0318 (2)	−0.0885 (4)	0.8204 (6)	0.0286 (11)	
H5A	−0.0670	−0.1391	0.8547	0.034*	
C6	0.0929 (2)	−0.0430 (4)	0.8022 (6)	0.0209 (10)	
O2W	0.28936 (19)	0.1261 (3)	0.3682 (4)	0.0363 (9)	
H2WB	0.259 (2)	0.108 (4)	0.444 (5)	0.051 (19)*	
H2WA	0.312 (2)	0.182 (3)	0.408 (6)	0.042 (16)*	

### Atomic displacement parameters (Å^2^)

**Table:**

	*U*^11^	*U*^22^	*U*^33^	*U*^12^	*U*^13^	*U*^23^
Co1	0.0154 (5)	0.0195 (5)	0.0206 (5)	0.0011 (3)	0.000	0.000
O1	0.0205 (17)	0.0295 (17)	0.0274 (18)	−0.0008 (13)	0.0002 (13)	0.0101 (14)
O1W	0.029 (2)	0.0236 (18)	0.032 (2)	0.0111 (14)	−0.0028 (15)	−0.0049 (14)
O2	0.0291 (19)	0.0258 (18)	0.038 (2)	−0.0023 (14)	−0.0044 (15)	0.0131 (15)
N1	0.023 (2)	0.0189 (18)	0.024 (2)	0.0013 (15)	−0.0020 (15)	0.0079 (15)
N2	0.033 (2)	0.023 (2)	0.036 (2)	−0.0045 (17)	−0.0012 (18)	0.0059 (18)
N3	0.033 (2)	0.027 (2)	0.035 (2)	−0.0027 (17)	0.0033 (18)	0.0082 (18)
N4	0.023 (2)	0.0206 (19)	0.027 (2)	−0.0040 (16)	−0.0012 (16)	0.0039 (16)
C1	0.028 (3)	0.019 (2)	0.019 (2)	0.0023 (19)	−0.0032 (19)	−0.0003 (18)
C2	0.022 (2)	0.018 (2)	0.020 (2)	−0.0037 (18)	−0.0049 (18)	0.0002 (18)
C3	0.028 (3)	0.023 (2)	0.024 (3)	0.0014 (19)	−0.0023 (19)	−0.0004 (19)
C4	0.019 (2)	0.034 (3)	0.029 (3)	0.0030 (19)	−0.0016 (19)	−0.003 (2)
C5	0.022 (3)	0.031 (3)	0.033 (3)	−0.008 (2)	0.002 (2)	−0.003 (2)
C6	0.019 (2)	0.022 (2)	0.021 (2)	−0.0030 (18)	−0.0032 (18)	0.0008 (19)
O2W	0.035 (2)	0.042 (2)	0.031 (2)	−0.0088 (17)	0.0054 (16)	−0.0055 (16)

### Geometric parameters (Å, °)

**Table:**

Co1—O1	2.095 (3)	N3—N4	1.362 (5)
Co1—O1^i^	2.095 (3)	N4—C6	1.365 (5)
Co1—O1W	2.102 (3)	N4—C5	1.382 (6)
Co1—O1W^i^	2.102 (3)	C1—C2	1.516 (6)
Co1—N1	2.129 (4)	C2—C3	1.378 (6)
Co1—N1^i^	2.129 (4)	C2—C6	1.424 (6)
O1—C1	1.286 (5)	C3—C4	1.432 (6)
O1W—H1WB	0.825 (18)	C3—H3A	0.9300
O1W—H1WA	0.842 (19)	C4—C5	1.353 (6)
O2—C1	1.242 (5)	C4—H4A	0.9300
N1—N2	1.342 (5)	C5—H5A	0.9300
N1—C6	1.351 (5)	O2W—H2WB	0.835 (19)
N2—N3	1.318 (5)	O2W—H2WA	0.842 (19)
			
O1—Co1—O1^i^	90.89 (16)	N2—N3—N4	106.0 (3)
O1—Co1—O1W	89.66 (13)	N3—N4—C6	108.1 (4)
O1^i^—Co1—O1W	176.49 (12)	N3—N4—C5	126.8 (4)
O1—Co1—O1W^i^	176.49 (12)	C6—N4—C5	125.1 (4)
O1^i^—Co1—O1W^i^	89.66 (13)	O2—C1—O1	125.0 (4)
O1W—Co1—O1W^i^	89.99 (18)	O2—C1—C2	117.0 (4)
O1—Co1—N1	83.91 (12)	O1—C1—C2	117.9 (4)
O1^i^—Co1—N1	95.17 (12)	C3—C2—C6	115.1 (4)
O1W—Co1—N1	88.33 (13)	C3—C2—C1	124.0 (4)
O1W^i^—Co1—N1	92.58 (13)	C6—C2—C1	120.8 (4)
O1—Co1—N1^i^	95.17 (12)	C2—C3—C4	123.8 (4)
O1^i^—Co1—N1^i^	83.91 (12)	C2—C3—H3A	118.1
O1W—Co1—N1^i^	92.58 (13)	C4—C3—H3A	118.1
O1W^i^—Co1—N1^i^	88.33 (13)	C5—C4—C3	119.6 (4)
N1—Co1—N1^i^	178.70 (19)	C5—C4—H4A	120.2
C1—O1—Co1	136.2 (3)	C3—C4—H4A	120.2
Co1—O1W—H1WB	120 (3)	C4—C5—N4	116.8 (4)
Co1—O1W—H1WA	115 (3)	C4—C5—H5A	121.6
H1WB—O1W—H1WA	109 (2)	N4—C5—H5A	121.6
N2—N1—C6	105.8 (3)	N1—C6—N4	108.0 (4)
N2—N1—Co1	130.3 (3)	N1—C6—C2	132.4 (4)
C6—N1—Co1	122.3 (3)	N4—C6—C2	119.6 (4)
N3—N2—N1	112.1 (3)	H2WB—O2W—H2WA	107 (3)
			
O1^i^—Co1—O1—C1	123.8 (4)	O2—C1—C2—C3	−2.7 (6)
O1W—Co1—O1—C1	−59.7 (4)	O1—C1—C2—C3	178.3 (4)
O1W^i^—Co1—O1—C1	25 (2)	O2—C1—C2—C6	173.1 (4)
N1—Co1—O1—C1	28.7 (4)	O1—C1—C2—C6	−5.9 (6)
N1^i^—Co1—O1—C1	−152.2 (4)	C6—C2—C3—C4	−1.1 (6)
O1—Co1—N1—N2	174.9 (4)	C1—C2—C3—C4	174.9 (4)
O1^i^—Co1—N1—N2	84.5 (4)	C2—C3—C4—C5	−1.7 (7)
O1W—Co1—N1—N2	−95.3 (4)	C3—C4—C5—N4	3.2 (6)
O1W^i^—Co1—N1—N2	−5.3 (4)	N3—N4—C5—C4	176.0 (4)
N1^i^—Co1—N1—N2	129.7 (4)	C6—N4—C5—C4	−2.1 (7)
O1—Co1—N1—C6	−21.2 (3)	N2—N1—C6—N4	0.4 (5)
O1^i^—Co1—N1—C6	−111.5 (3)	Co1—N1—C6—N4	−166.9 (3)
O1W—Co1—N1—C6	68.6 (3)	N2—N1—C6—C2	178.1 (5)
O1W^i^—Co1—N1—C6	158.6 (3)	Co1—N1—C6—C2	10.8 (7)
N1^i^—Co1—N1—C6	−66.4 (3)	N3—N4—C6—N1	−1.1 (5)
C6—N1—N2—N3	0.4 (5)	C5—N4—C6—N1	177.4 (4)
Co1—N1—N2—N3	166.3 (3)	N3—N4—C6—C2	−179.1 (4)
N1—N2—N3—N4	−1.1 (5)	C5—N4—C6—C2	−0.7 (6)
N2—N3—N4—C6	1.3 (4)	C3—C2—C6—N1	−175.3 (4)
N2—N3—N4—C5	−177.1 (4)	C1—C2—C6—N1	8.6 (7)
Co1—O1—C1—O2	162.2 (3)	C3—C2—C6—N4	2.2 (6)
Co1—O1—C1—C2	−18.9 (6)	C1—C2—C6—N4	−173.9 (4)

Symmetry codes: (i) −*x*+1/2, −*y*, *z*.

### Hydrogen-bond geometry (Å, °)

**Table:**

*D*—H···*A*	*D*—H	H···*A*	*D*···*A*	*D*—H···*A*
O1W—H1WB···O2^ii^	0.83 (2)	1.95 (2)	2.763 (4)	168 (4)
O1W—H1WA···O2W^iii^	0.84 (2)	1.94 (2)	2.776 (5)	170 (5)
O2W—H2WB···O1	0.84 (2)	2.04 (3)	2.845 (4)	163 (4)
O2W—H2WA···N2^iv^	0.84 (2)	2.15 (2)	2.981 (5)	171 (4)

Symmetry codes: (ii) *x*, −*y*+1/2, −*z*+3/2; (iii) *x*, *y*, *z*+1; (iv) −*x*+1/2, *y*+1/2, −*z*+3/2.
